# VitelloTag: a tool for high-throughput cargo delivery into oocytes

**DOI:** 10.1242/dev.202857

**Published:** 2024-09-14

**Authors:** D. Nathaniel Clarke, Akshay Kane, Margherita Perillo, Christopher J. Lowe, S. Zachary Swartz

**Affiliations:** ^1^ Department of Biology, Massachusetts Institute of Technology, Cambridge, MA 02142, USA; ^2^ The Eugene Bell Center for Regenerative Biology and Tissue Engineering, Marine Biological Laboratory, 7 MBL Street, Woods Hole, MA 02543-1015, USA; ^3^ Hopkins Marine Station, Stanford University, Pacific Grove, CA 93950, USA

**Keywords:** CRISPR/Cas9, Oocyte delivery, Vitellogenesis, Gene editing, Transgenesis

## Abstract

Delivering molecular tools into oocytes is essential for developmental and reproductive biology. Microinjection, the conventional method, is equipment intensive, often technically challenging and has a low yield, and is impractical in species with delicate oocytes or restricted spawning seasons. To overcome these limitations, we developed VitelloTag, a cost-effective, high-throughput system using vitellogenin-derived fusion proteins to enable efficient cargo delivery via receptor-mediated endocytosis. We demonstrate its utility by delivering Cas9/sgRNA complexes in two distantly related species for gene knockout.

## Teaser

**Summary:** VitelloTag, a method for transporting protein cargo into oocytes, facilitates microinjection-free CRISPR/Cas9 genome editing in multiple species.

## INTRODUCTION

CRISPR/Cas9 technology has revolutionized functional studies not only in established model systems (Friedland et al., 2013; Gratz et al., 2013; Hwang et al., 2013; Wang et al., 2013), but also in a range of emerging research organisms (Munro et al., 2023; Oulhen et al., 2022; Perillo et al., 2023; Pickett and Zeller, 2018). Nonetheless, a major bottleneck in many experiments is the delivery of CRISPR reagents into oocytes for the germline transmission of mutations and analysis of mutant phenotypes in F0 embryos. Typically, this involves microinjecting gene-editing reagents directly into the oocyte or fertilized zygote. However, microinjection is often a substantial or insurmountable technical hurdle in many species: oocytes are often too fragile to withstand the injection process or possess tough outer chorions; some species, like many species of coral that spawn only one night per year, can have very limited spawning seasons; making gene manipulation by microinjection impractical. Moreover, microinjection often has a steep learning curve and requires specialized training, involves expensive equipment, and is labor intensive.

**Fig. 1.**
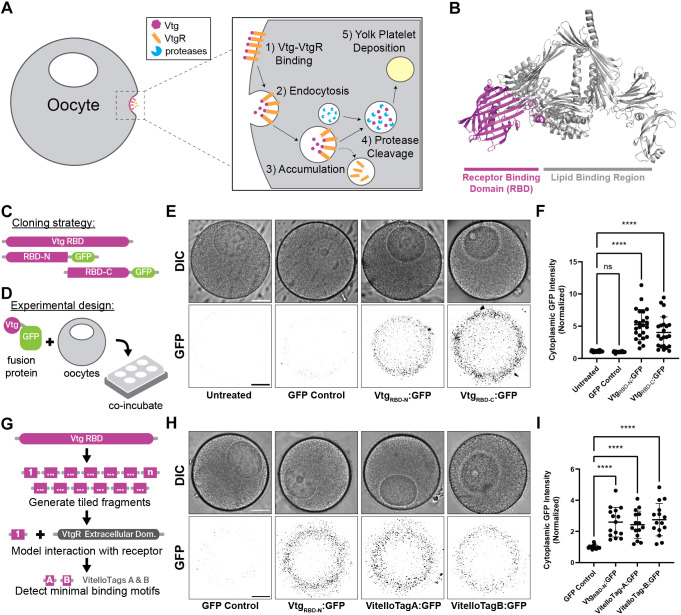
**Vitellogenin fusion tags enable efficient trafficking of proteins into oocytes.** (A) Schematic of the endogenous vitellogenesis pathway. Egg yolk proteins produced in somatic tissues are taken up by developing oocytes through receptor-mediated endocytosis. After internalization, Vtg accumulates in pre-lysosomal endocytic compartments, and is then processed into two subunits that remain in mature endocytic vesicles until needed. (B) Structure of lamprey Vtg (PDB:1LSH); receptor binding domain is highlighted (magenta). (C) Schematic of cloning strategy used in this study. (D) *In vitro* oocyte transport assay. Vtg:GFP fusions are co-incubated with live oocytes overnight, and then screened for GFP content. (E) DIC (top) and fluorescence (bottom) micrographs of oocytes treated with Vtg:GFP fusion proteins. GFP images are inverted grayscale. Scale bars: 50 µm. (F) Quantification of cytoplasmic GFP intensities from E (normalized relative to untreated oocytes). Dots represent single oocytes of untreated control (*n*=24, two independent experiments), GFP-only control (*n*=24, two independent experiments), Vtg_RBD-N_:GFP (*n*=24, two independent experiments) and Vtg_RBD-C_:GFP (*n*=23, 2 independent experiments). Significance was determined by one-way ANOVA (****P<0.0001). Data are mean±s.d. (G) Workflow for computational identification of minimal binding fragments. (H) DIC (top) and fluorescence (bottom) micrographs of oocytes treated with VitelloTag:GFP fusion proteins. GFP images are inverted grayscale. Scale bars: 50 µm. (I) Quantification of cytoplasmic GFP intensities from H (normalized relative to GFP-only control oocytes). Dots represent single oocytes of GFP-only control (*n*=15, two independent experiments), Vtg_RBD-N_:GFP (*n*=15, two independent experiments), VitelloTagA:GFP (*n*=15, two independent experiments) and VitelloTagB:GFP (*n*=15, two independent experiments). Significance was determined by one-way ANOVA (****P<0.0001). Data are mean±s.d.

To overcome these limitations, we developed a cell-penetrating technique using a natural receptor-mediated endocytic pathway to facilitate specific and efficient trafficking into oocytes. Our approach focuses on harnessing vitellogenesis: the process of yolk accumulation in growing oocytes (Fig. 1A). We targeted Vitellogenin (Vtg), a widely conserved yolk protein that is found in all metazoan phyla except ctenophores (Byrne et al., 1989; Lebouvier et al., 2022), to develop a versatile tool applicable in a phylogenetically diverse range of species. The N-terminal region of Vtg contains a receptor-binding domain (RBD) that specifically interacts with the Vitellogenin receptor (VtgR) to facilitate uptake (Fig. 1B) (Li et al., 2003). We hypothesized that the Vtg RBD could be used to generate a fusion tag sufficient to enable trafficking of protein cargos into oocytes.

## RESULTS AND DISCUSSION

Using the sea star *Patiria miniata* as a representative model widely used for reproductive and developmental biology studies, we tested whether the RBD region of *Pm* Vtg1 was sufficient to transport protein cargo into oocytes. To identify required RBD regions, we recombinantly fused the N- and C-terminal halves of the RBD with Superfolder GFP (sfGFP) and a nuclear localization sequence (NLS) (Fig. 1C), and tested their endocytosis into oocytes *in vitro* (Fig. 1D). Both Vtg fragments were successfully endocytosed with equivalent efficiency (Fig. 1E,F). GFP-containing vesicles predominantly localized to the cell periphery. Consistent with this acting through the vitellogenesis pathway, uptake required dynamin-mediated endocytosis (Fig. S1A,B) (Macia et al., 2006). Because Vtg is typically targeted to acidified vesicles, endosome escape is crucial for effective cargo delivery into the cytoplasm. We found that the endosome escape reagent chloroquine induced the release of Vtg-sfGFP, confirming their import into the nucleus via their NLS (Fig. S1C,D). Importantly, chloroquine was well tolerated during development and did not disrupt embryogenesis (Fig. S2). Collectively, these results demonstrate the potential of Vtg protein fusions for cargo delivery *en masse* in simple *in vitro* oocyte cultures.

Our observations indicated that both halves of the *Pm* Vtg1 RBD contain binding motifs that are sufficient for oocyte transport. To identify these sequences computationally, we used a modelling approach with AlphaFold Multimer to predict interactions between short fragments of *Pm* Vtg1 and *Pm* VtgR (Jumper et al., 2021; Mirdita et al., 2022) (Fig. 1G). By focusing on fragments predicted to have the highest number of interactions with *Pm* VtgR (Fig. S3A) and using a per-residue interaction score (Fig. S3B) in conjunction with structural modeling (Fig. S3C,D), we identified two short (∼10-15 amino acid) ‘best hit’ motifs in the N- and C-terminal halves. To assess whether these minimal sequences, which we termed VitelloTag A (residues 28-36: SITIHRNTP) and VitelloTag B (residues 197-209: LMTILNVTKVRDL), could serve as minimal oocyte transport tags, we cloned sfGFP fusions and subjected them to the same *in vitro* oocyte transport assay. Notably, sfGFP fusions of VitelloTag A and B showed equivalent uptake efficiency to the larger parent fragments (Fig. 1H,I).

**Fig. 2.**
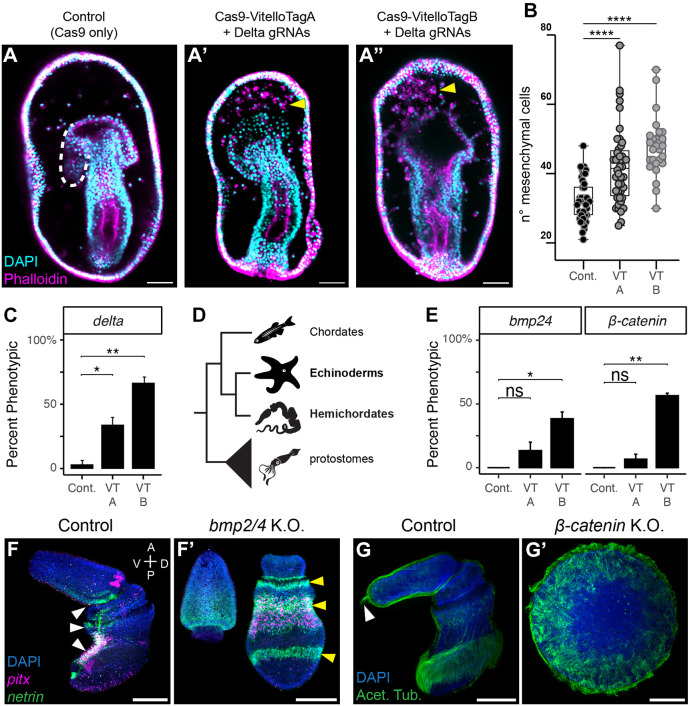
**VitelloTag-Cas9 enables injection-free genome editing in multiple animal phyla.** (A) Embryos treated with VitelloTag-A and VitelloTag-B:Cas9 show the characterized phenotype of increased mesenchymal cells (yellow arrowheads) and failure to form coelomic tubes (dashed white line, control). (B) Number of mesenchymal cells in 2-day late gastrulae. Dots represent single embryos, controls were treated with VitelloTag-A or VitelloTag-B:Cas9 without gRNAs (*n*=42, three independent experiments). VitelloTag-A:Cas9+*delta* gRNAs (*n*=44, three independent experiments, ****P<0.0001). VitelloTag-B:Cas9+*delta* gRNAs (*n*=27, three independent experiments, ****P<0.0001). Data are mean±s.e.m. Statistical significance was assessed using a two-sided unpaired Student's *t*-test. The median is the middle line, the box represents the 25th and 75th percentiles, and whiskers indicate the minimum and maximum data range. (C) Quantification of phenotypic embryos in *Patiria* experiments. Phenotypic embryos were defined as having mesenchyme cell numbers at least two standard deviations higher than the mean value for control embryos. Significance was determined using an unpaired Student's *t*-test (*P<0.05, **P<0.01). Data are mean±s.d. (D) Phylogeny of bilaterian phyla; groups examined in this study are highlighted in bold. (E) Quantification of phenotypic embryos in *Saccoglossus* experiments. Significance was determined using an unpaired Student's *t*-test (*P<0.05, **P<0.01). Data are mean±s.d. (F,F′) *Saccoglossus* embryos treated with VitelloTag-B:Cas9 and *bmp2/4* gRNAs show the characteristic phenotype of radialization of the dorsal-ventral body axis. Marker genes typically expressed on the ventral side (F; *pitx* and *netrin*, white arrowheads) become circumferential stripes in *bmp2/4* F0 mutants (F′, yellow arrowheads). (G) *Saccoglossus* embryos treated with VitelloTag-B:Cas9 and *β-catenin* gRNAs show the characteristic phenotype of anteriorization of the anterior-posterior body axis. Elongated sensory cilia typically restricted to the far anterior (G, white arrowhead) cover the entire body axis (G′) and the embryo fails to gastrulate.

To determine the utility of VitelloTags for transporting gene editing reagents, we generated NLS-eSpCas9(1.1) fusion proteins with each VitelloTag. We first tested for successful internalization of the VitelloTagCas9 constructs using immunostaining. The constructs were successfully internalized into the oocyte cytoplasm and showed comparable nuclear localization to Cas9 mRNA-injected oocytes, along with the anticipated endosomal signal near the cortex due to its endocytosis (Fig. S4). We then tested both Vtg-Cas9 proteins for the generation of F0 knockout embryos by targeting the *delta* locus (which has been extensively studied and shows a clear phenotype) with three synthetic sgRNAs (used together). By 3 days post fertilization, *delta* knockout embryos displayed strong defects consistent with previously published *delta* knockdown and knockout phenotypes in *P. miniata* (Fig. 2A) (Cary et al., 2020; Perillo et al., 2023). Specifically, we scored the number of mesenchyme cells, which is significantly increased compared with Cas9 alone controls in both VitelloTagA:Cas9 and VitelloTagB:Cas9 (Fig. 2B). The frequency of phenotypic embryos was 32% and 63%, respectively (see Methods; Fig. 2C). We also performed HCR *in situ* hybridizations against *gcm*, the overexpression of which is a marker for *delta* knockout in *P. miniata* embryos (Zueva and Hinman, 2023 preprint). We saw a greater number of cells expressing *gcm* when compared with the untreated control embryos (Fig. S5). Genotyping analysis of individual embryos by TIDE spectral decay, a method that determines the percentage of sequences amplified from a genomic locus that contain an indel (‘total efficiency’) (Brinkman et al., 2014), confirmed disruption of the *delta* allele (30.4% and 43.9%, respectively, for VitelloTag-Cas9 versus microinjection of Cas9 mRNA) (Fig. S6). VitelloTag:Cas9 is thus an effective reagent for high-throughput generation of knockout embryos, although knockout efficiency is mosaic, based on TIDE analysis and phenotypic penetrance.

To evaluate the potential of VitelloTag:Cas9 for genome editing in other more distantly related organisms, we next tested it in the hemichordate enteropneust *Saccoglossus kowalevskii* (Fig. 2D). As *Saccoglossus* is challenging to microinject, VitelloTag could significantly enhance the experimental possibilities in this organism. Our experiments showed that both VitelloTag A and B Cas9 fusions successfully induced indel mutations and knockout phenotypes in *Saccoglossus*, albeit with varying penetrance (10% and 48%, across two target genes, for VitelloTag A and B, respectively; Fig. 2E). VitelloTag-B:Cas9 was particularly effective, generating penetrant knockout phenotypes for two target genes, *bmp2/4* and *beta-catenin*, at frequencies approaching the efficiency seen in *P. miniata* (38% and 47%, for *bmp2/4* and *beta-catenin*, respectively. *n*=45 in three replicates of 15 embryos) (Fig. 2F,G). The observed phenotypes were consistent with zygotic gene knockout: embryos treated with gRNAs targeting *bmp2/4*, which is active after zygotic genome activation, phenocopied the previously published RNAi phenotype of radialization of the dorsal-ventral axis (Fig. 2F) (Lowe et al., 2006), whereas embryos treated with gRNAs targeting *beta-catenin*, which has both maternal and zygotic effects, displayed only the zygotic phenotype of anteriorization of the anterior-posterior axis (Fig. 2G, Movie 1) (Darras et al., 2011, 2018). These results represent the first knockout phenotypes in this important group of animals.

Our results establish the feasibility of cell-penetrant CRISPR/Cas9 genome editing in oocytes for comparative developmental studies. We show that VitelloTags enable high-throughput trafficking of active Cas9-sgRNA ribonucleoprotein complexes in two species, overcoming multiple limitations of standard microinjection protocols. Although similar strategies have been developed in arthropods (e.g. ReMOT Control in mosquitos; Chaverra-Rodriguez et al., 2018), they exploit non-conserved proteins, and are therefore limited in their cross-species utility. Thus, our results set the stage for broader use of vitellogenin-based transgenesis in diverse emerging model systems. We identify VitelloTag B as a potential cross-reactive oocyte transport tag that is effective in two distantly related species. Sequence conservation analysis indicates that VitelloTag B resides in a highly conserved region of the Vitellogenin receptor-binding domain (Fig. S7), which suggests that it may be functional in a broader array of organisms. Not only does Vitellotag potentially open up gene editing approaches to a wider phylogenetic range of animals, it may also facilitate functional studies in more remote and poorly equipped field stations where most biodiversity is accessible but microinjection is impractical. These simple methods also lend themselves well to undergraduate teaching laboratories. We note that this version of VitelloTag does not achieve complete knockout efficiency. This may be due to incomplete endosome escape or to the short half-life of Cas9 protein in animal cells, two parameters that will require further work to optimize (Kim et al., 2014). Importantly, this system may be potentially adapted for the delivery of nucleic acids and other proteins, further expanding the functional tools available in non-canonical research organisms.

## MATERIALS AND METHODS

### Research organisms

Wild-caught Bat Stars (*Patiria miniata*) were obtained from South-Coast Bio (San Diego, CA, USA) and Monterey Abalone Company (Monterey, CA, USA). The age of the animals cannot be determined. Individuals were randomly selected for oocyte and/or sperm isolation. Acorn worms (*Saccoglossus kowalevskii*) were field-collected in Waquoit Bay (Falmouth, MA, USA). For *Saccoglossus*, natural spawning of oocytes and isolation of sperm was performed as previously described (Lowe et al., 2004).

### Gonad extraction

Intact ovary and testis fragments were surgically extracted using forceps through small slits on the oral side of an arm of the sea star. Ovary pieces were kept in sterile seawater containing 10 μg/ml trimethoprim and 50 μg/ml sulfamethoxazole at 15°C. The ovary fragments were transferred to fresh seawater with antibiotics every other day and only fresh oocytes teased from the ovary were used for experiments (as described by Swartz et al., 2019).

### Oocyte and embryo culture with VitelloTag

Isolated oocytes were cultured in sterile seawater plus antibiotics for a maximum of 24 h. In all experiments involving Vtg:GFP uptake, treatment and control groups were incubated with 1 μg/ml Vtg:GFP and GFP respectively, overnight. For endosomal escape trials, oocytes were incubated with defined concentrations of chloroquine overnight in addition to Vtg:GFP. Dynasore trials were incubated with defined concentrations of Dynasore in addition to Vtg:GFP, alongside vehicle controls (DMSO, 1:1000 dilution). For all experiments, oocytes were kept at 15°C.

For chloroquine toxicity assays, oocytes were incubated overnight (16-18 h) at 15°C in varying concentrations of chloroquine. On the next day, the oocytes were stimulated with 1-methyladenine at a final concentration of 10 μM to induce meiotic resumption. For fertilization, extracted sperm was added at a 1:500,000 dilution before emission of the first polar body. Fertilized oocytes were transferred to fresh seawater without antibiotics and kept at 15°C.

### Sample preparation and imaging

For live imaging, oocytes were mounted on a glass dish in ∼10 μl of seawater under a glass coverslip. For analysis of mesenchyme cells, *P. miniata* embryos were fixed in 2% paraformaldehyde (PFA)/sea water (SW) for 30 min, washed in PBST and stained with phalloidin (1:300) and DAPI. For HCR *in situ* hybridization, *P. miniata* embryos were fixed in 4% PFA/MOPS Fixative buffer and then processed with an echinoderm HCR protocol published previously (Formery et al., 2023). For Cas9 immunofluorescence, *P. miniata* oocytes were fixed in 2% PFA/SW for 1 h, washed with PBST and methanol, and then stained using anti-Cas9 antibody (Sigma, SAB4200701-25UL; 1:500 dilution) and Hoechst. *S. kowalevskii* embryos were fixed in 4% formaldehyde for 1 h, as described previously (Lowe et al., 2004). Embryos were then either stained with anti-acetylated Tubulin antibody (Sigma, T7451; 1:500 dilution) and DAPI, or prepared for fluorescent *in situ* hybridization using the HCR method (Molecular Instruments) following the manufacturer's instructions.

### Image quantification and statistical analysis

Statistical analyses were performed using Prism (10.3.0 GraphPad Software). Details of the statistical test used, sample size and significance values are included in figure legends. Images comparing the same signal across groups are scaled linearly and equivalently.

Quantification of nuclear and cytoplasmic signal intensity was performed using Fiji/ImageJ (Schindelin et al., 2012). A maximum intensity *z*-projection of the first 31 slices of each image was performed to measure cytoplasmic GFP signal intensity (Fig. 1E,H and Fig. S1A). Projections were then background subtracted with the rolling ball (radius=50 pixels) and sliding paraboloid function. A 120 μm circular region of interest was used to measure the pixel intensity in the cytoplasm. Quantification was obtained using the *RawIntDen* parameter in the *Measure* function. To measure pixel intensity in the nucleus (Fig. S1C), a single *z*-slice that was captured inside the nucleus of the oocyte was chosen. A 50 μm circular region of interest was placed in the nucleus, another 50 μm circular region of interest was placed in the background. Pixel intensities of both regions were obtained using the *RawIntDen* parameter in the *Measure* function. The *RawIntDen* value of background was subtracted from that of the nucleus to obtain the final quantification of pixel intensity for a given image. For measuring Cas9 nuclear signal (Fig. S4), shallow *z*-projections of 15 slices each were taken for all samples. These projections then underwent background subtraction with the rolling ball (radius=50 pixels) and sliding paraboloid function. A 50 μm circular region of interest was then placed in the nucleus of each oocyte. Pixel intensities of Cas9 signal were obtained using the *RawIntDen* parameter in the *Measure* function. The ‘multi-point’ tool on ImageJ was used to count the number of individual *gcm*-positive cells in an embryo (Fig. S5).

### Protein structure prediction and interaction analysis

Overlapping tiled sequence fragments of Pm Vtg1 RBD (residues 1-315) were generated using a 50 amino acid sliding window with a step size of 25 amino acids, and then used as input for AlphaFold Multimer along with the N-terminal region of Pm VtgR (LDL domains 1-3, residues 72-190). Interactions between chains within 4 Angstroms were counted per fragment, and then weighted by multiplication with the average pLDDT score across the fragment. Per-residue normalized interactions were calculated as the average interaction score between overlapping fragments, per residue position. The best peaks from both N- and C-terminal halves were targeted for in-depth structural analysis, which was then used to pinpoint contiguous regions with a high number of predicted protein-protein interactions.

### Molecular cloning

Amplicons were generated by PCR using Q5 proof-reading polymerase (NEB) using primers reported in Table S1. Constructs were then generated using Gibson Assembly Master Mix (NEB) following the manufacturer's instructions.

### Protein expression and purification

Vtg-sfGFP fusion proteins were expressed as 6× His-tagged constructs in BL21(DE3) *E. coli*. Protein expression was induced with 0.1 mM IPTG overnight at 18°C. Vtg-Cas9 fusions that were toxic to BL21 cells were produced using BL21-AI *E. coli* cells. Here, protein expression was induced with 0.1% Arabinose overnight at 18°C. Proteins were purified from 3 l of bacterial culture with Ni-NTA Agarose Beads (GoldBio) and eluted with imidazole (Sigma). Purity was confirmed by SDS-PAGE analysis.

### CRISPR/Cas9 experiments

#### Patiria CRISPR/Cas9 with VitelloTag:Cas9

gRNAs were ordered from Synthego and resuspended in nuclease-free water at a concentration of 100 µM. VitelloTag:Cas9 proteins were prepared as described above. A step-by-step protocol is provided in the supplementary Materials and Methods. In brief, a solution of VitelloTagA:Cas9 (7 mg/ml) or VitelloTagB:Cas9 (4 mg/ml) was used 1:250, plus 0.8 µl each of 100 µM *delta* gRNAs 198, 248 and 519 [target sequences as in Perillo et al. (2023) and are reported in Table S2] and NEB buffer r3.1 in a final volume of 20 μl was left for 20 min at 25°C followed by 5 min at 37°C to form a Cas9-gRNA ribonucleoprotein (RNP) complex. The RNP solution was then added to oocytes together with 25 µM chloroquine and antibiotics at a dilution of 1:1000 in 500 µl of filtered sea water. Oocytes were kept at 15°C overnight, and then matured and fertilized. Fertilized oocytes were washed with antibiotic-free fresh filtered seawater to remove chloroquine, antibiotics and VitelloTag:Cas9. After gastrulation, the *delta* knockout phenotype was scored by counting the number of mesenchyme cells present in the embryo; phenotypic embryos were defined as those having cell numbers at least two standard deviations higher than the mean value for control embryos.

#### Individual larva genotyping and TIDE spectral decay analyses

Individual larvae were genotyped with PCRs of individual embryos that were sequenced and used for TIDE analysis using primers oZS1032 and oZS1033. Sequence trace files for CRISPR-Cas9 edited and Cas9 controls were analyzed using differential sequence-trace chromatograph analysis with TIDE [Tracking of Indels by Decomposition (V. 3.3.0)] (Brinkman et al., 2014).

#### *Saccoglossus* CRISPR/Cas9 with VitelloTag:Cas9

CRISPR/Cas9 RNP complexes were formed by mixing sgRNAs (two per target gene) and VitelloTag-Cas9 at a 1:1:1 ratio at a concentration of 1.8 µM, followed by incubation at 25°C for 20 min, then 37°C for 5 min. Unfertilized *Saccoglossus* oocytes were collected in 500 µl of filtered seawater, then incubated with RNPs diluted 1:50 to a final concentration of 36 nM at 22°C in the presence of 25 µM chloroquine. After 4 h, eggs were fertilized with one drop of diluted sperm solution, and kept in the same solution containing RNPs and escape reagent overnight for ∼12 h. Embryos were screened for phenotypes at 36 h and 72 h, for *β-catenin* and *bmp2/4*, respectively. Penetrance was quantified as the percentage of embryos displaying an unambiguous knockout phenotype.

### Resource availability

Plasmids for bacterial production of VitelloTag proteins will be made available for academic use on Addgene.

## Supplementary Material



10.1242/develop.202857_sup1 Supplementary information
