# Supplementary material for: VitelloTag: a tool for high-throughput cargo delivery into oocytes
Source: Development. 2024 Sep 14;151(20):dev202857. doi: 10.1242/dev.202857 (PMC11423919; doi:10.1242/dev.202857)
Supplement: Supplementary information [file develop-151-202857-s1.pdf]

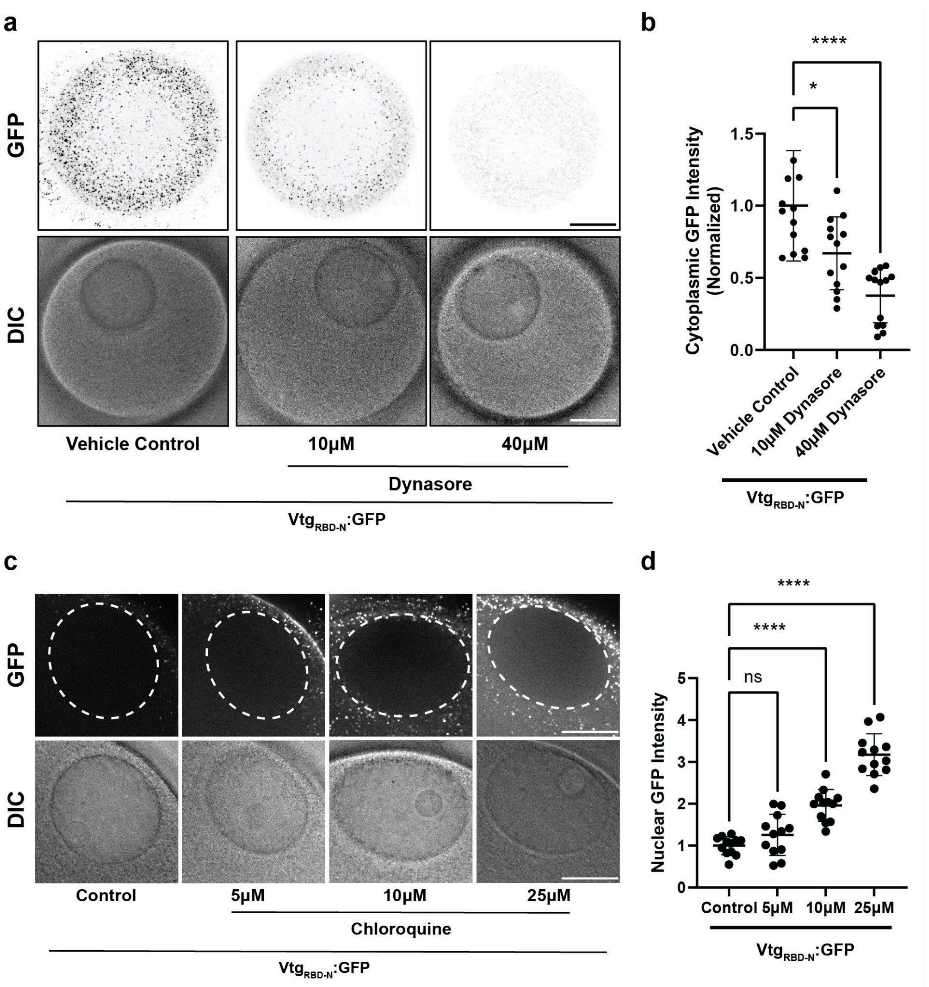

Fig. S1. Vitellogenin fusion proteins are internalized via dynamin-mediated endocytosis

a. Incubation with increasing concentrations of the Dynamin inhibitor, Dynasore, reduces uptake of Vtg:GFP. GFP images are z-projections. Scale bar is 50 μm.
b. Quantification of cytoplasmic GFP intensities in (a) (normalized relative to control oocytes). Dots represent single Vtg_{RBD-N}:GFP soaked oocytes treated with vehicle control (DMSO) (n=13, 2 independent experiments),10μM Dynasore (n=13, 2 independent experiments), and 40μM Dynasore (n=13, 2 independent experiments). Significance determined by one-way ANOVA, ****p< 0.0001. Error bars represent mean and standard deviation.
c. Incubation with increasing concentrations of the endosome escape reagent, chloroquine, increases nuclear intensity of Vtg:GFP (diffuse signal within region bounded by dashed line). Images shown are single slice zoom views of the germinal vesicle (oocyte nucleus, indicated with dashed lines). Scale bar is 50 μm.
d. Quantification of nuclear GFP intensities in (c) (normalized relative to control oocytes). Dots represent single Vtg_{RBD-N}:GFP soaked oocytes from a control group (n=12, 2 independent experiments), and groups treated with 5μM (n=12, 2 independent experiments), 10μM (n=12, 2 independent experiments), and 25μM Chloroquine (n=12, 2 independent experiments). Significance determined by one-way ANOVA, ****p< 0.0001. Error bars represent mean and standard deviation.

Development • Supplementary information

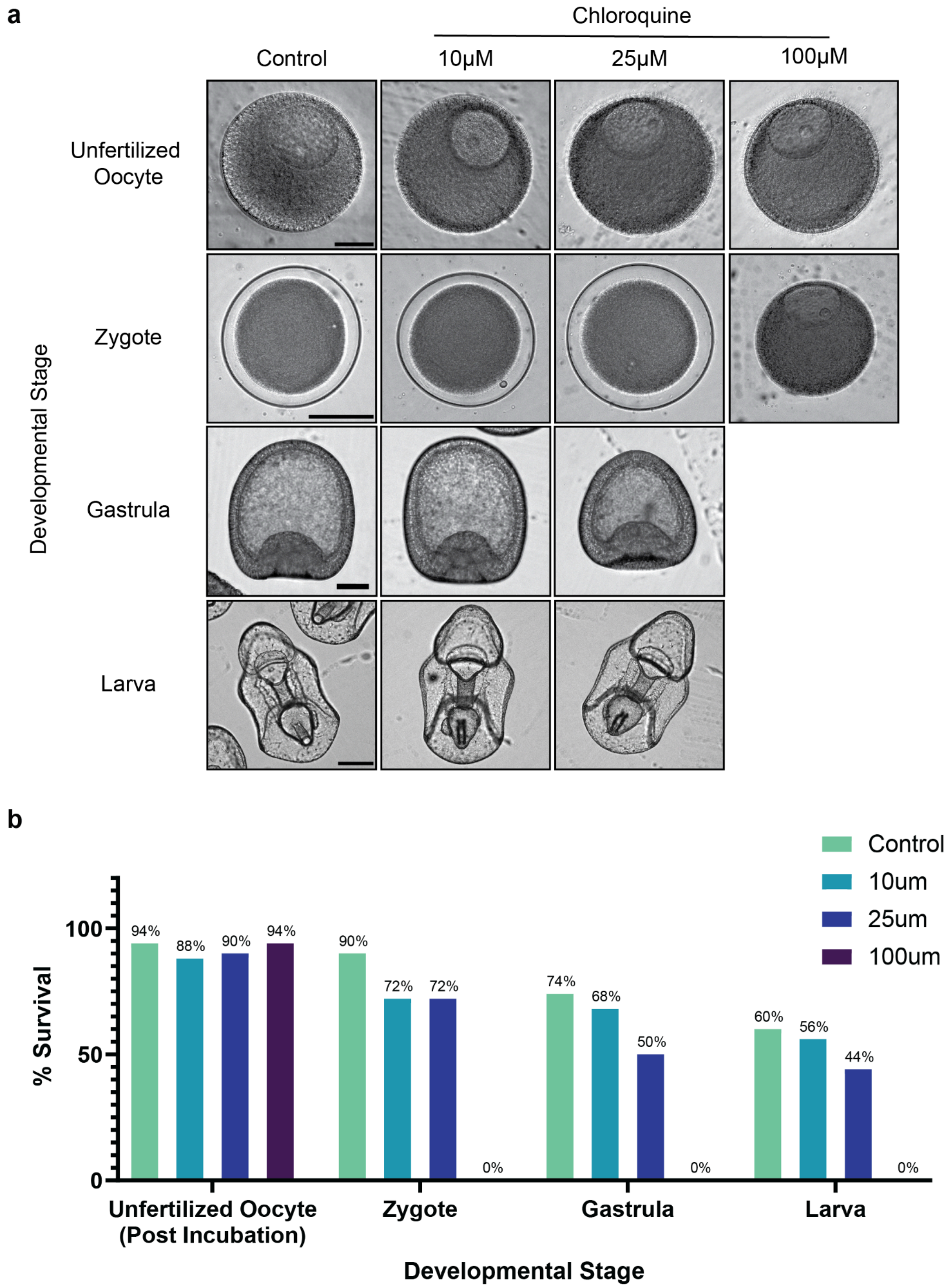

Fig. S2. Chloroquine is not toxic to embryonic development at moderate doses in *P. miniata*

a. Micrographs of oocytes and embryos treated with increasing concentrations of chloroquine. Scale bars are 50 μm, except in larva images, where scale bar is 150 μm.
b. Quantification of survival of embryos treated with chloroquine (n=50 oocytes, assessed longitudinally through developmental stages after fertilization).

Development • Supplementary information

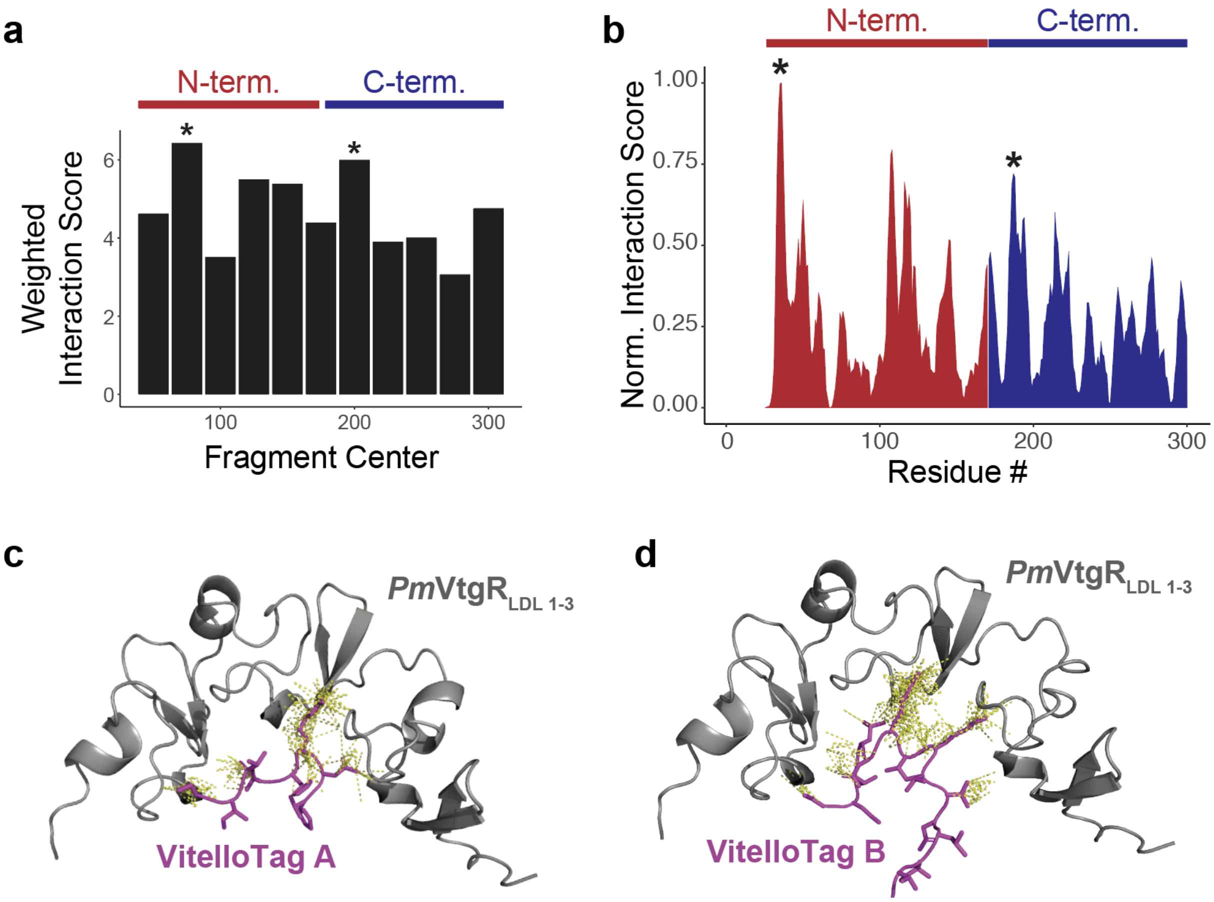

Fig. S3. Identification of minimal VitelloTag sequences using structural prediction.

a. Quantification of average number of interactions between 50aa fragments of *Pm* Vtg1 with *Pm* VtgR. Asterisks denote fragments with highest interaction score in the N- and C-terminal halves of the Vtg RBD.

b. Quantification of interaction score per residue, averaged across residue positions from tiled 50aa Vtg fragments. Colors denote N- and C-terminal halves of the RBD, and asterisks indicate the highest peaks in interaction score within each half.

c, d. Structural predictions for minimal Vtg:VtgR interactions between VitelloTags A and B (magenta) and the N-terminal region of the VtgR extracellular domain (gray). Predicted protein-protein interactions within 4 angstroms are depicted as dashed yellow lines.

Development • Supplementary information

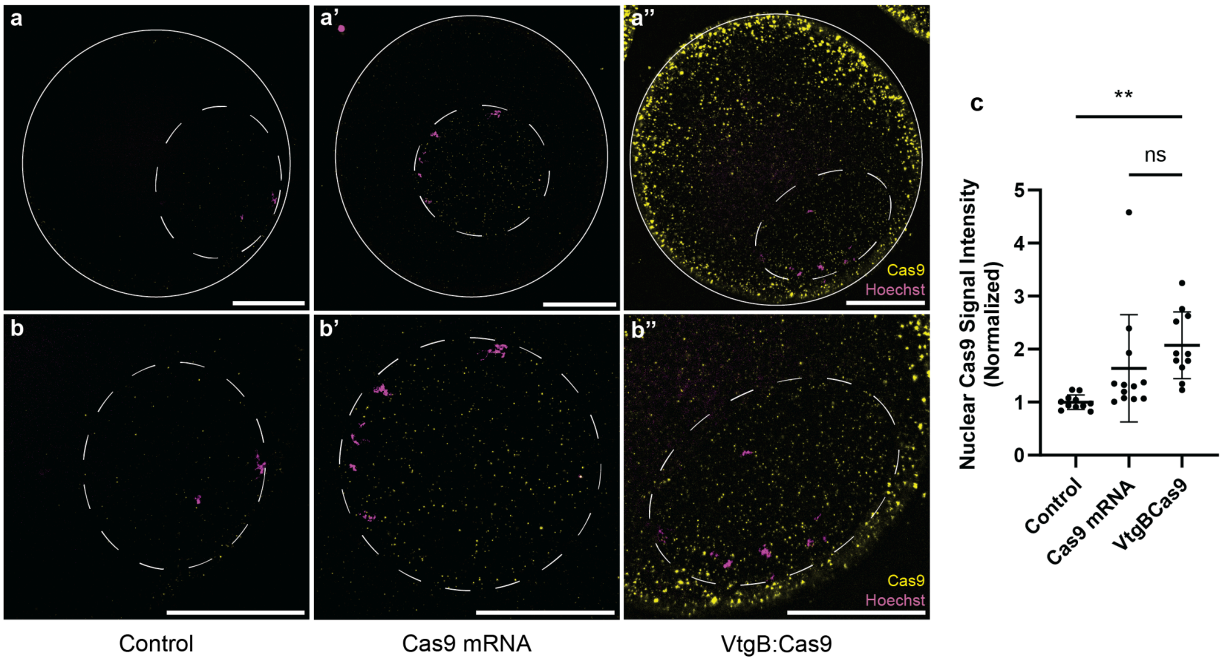

Fig. S4. VitelloTag:Cas9 localizes to the nucleus

a. Cas9 immunostaining of untreated (a), Cas9 mRNA injected (a'), and VitelloTagB:Cas9 soaked (a'') oocytes. Yellow is Cas9 signal and magenta is Hoechst (DNA). Solid line indicates oocyte cell surface, and dashed line indicates the oocyte germinal vesicle (nucleus). Images are shallow 15-slice z-projections. Scale bars are 50μM.
b. Cropped images of respective nuclei of oocytes from (a). Both Cas9 mRNA and VitelloTagB:Cas9 treated oocytes (b', b'') show comparable nuclearization of Cas9. This signal is absent in control oocytes (b). Yellow is Cas9 signal and magenta is Hoechst (DNA). Dotted line indicates the oocyte germinal vesicle (nucleus). Scale bars are 50μM.
c. Quantification of nuclear Cas9 signal intensity (normalized relative to untreated oocytes). Significance determined by one-way ANOVA test, ** $p = 0.002$. Error bars represent mean and standard deviation.

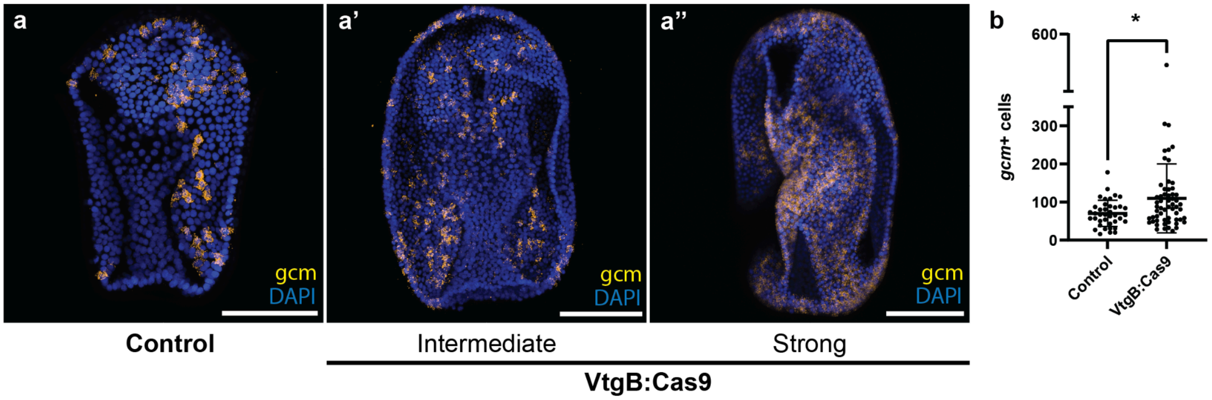

Fig. S5. VitelloTag:Cas9 knockout of *delta* increases the number of *gcm*-positive cells

a. *P. miniata* embryos (2dpf) treated with VitelloTag-B:Cas9 and *delta* gRNAs display an increase in *gcm* expressing cells. Z-projections of representative embryos with an intermediate (a') and strong (a'') phenotype are shown in comparison to a no gRNA-control embryo. Scale bars are 100µM.

b. Count of *gcm*+ cells in embryos. Dots represent single embryos, controls treated with VitelloTagB:Cas9 without gRNAs (n=39, 4 independent experiments), VitelloTagB:Cas9 + *delta* gRNAs (n=58, 4 independent experiments), * p = 0.0231. Y-axis break has been added at the 350 mark and resumes at 550 mark to include an extreme sample from the VitelloTag-Cas9 treated embryos. Statistical significance was assessed by a one-way ANOVA test. Error bars represent mean and standard deviation.

Development • Supplementary information

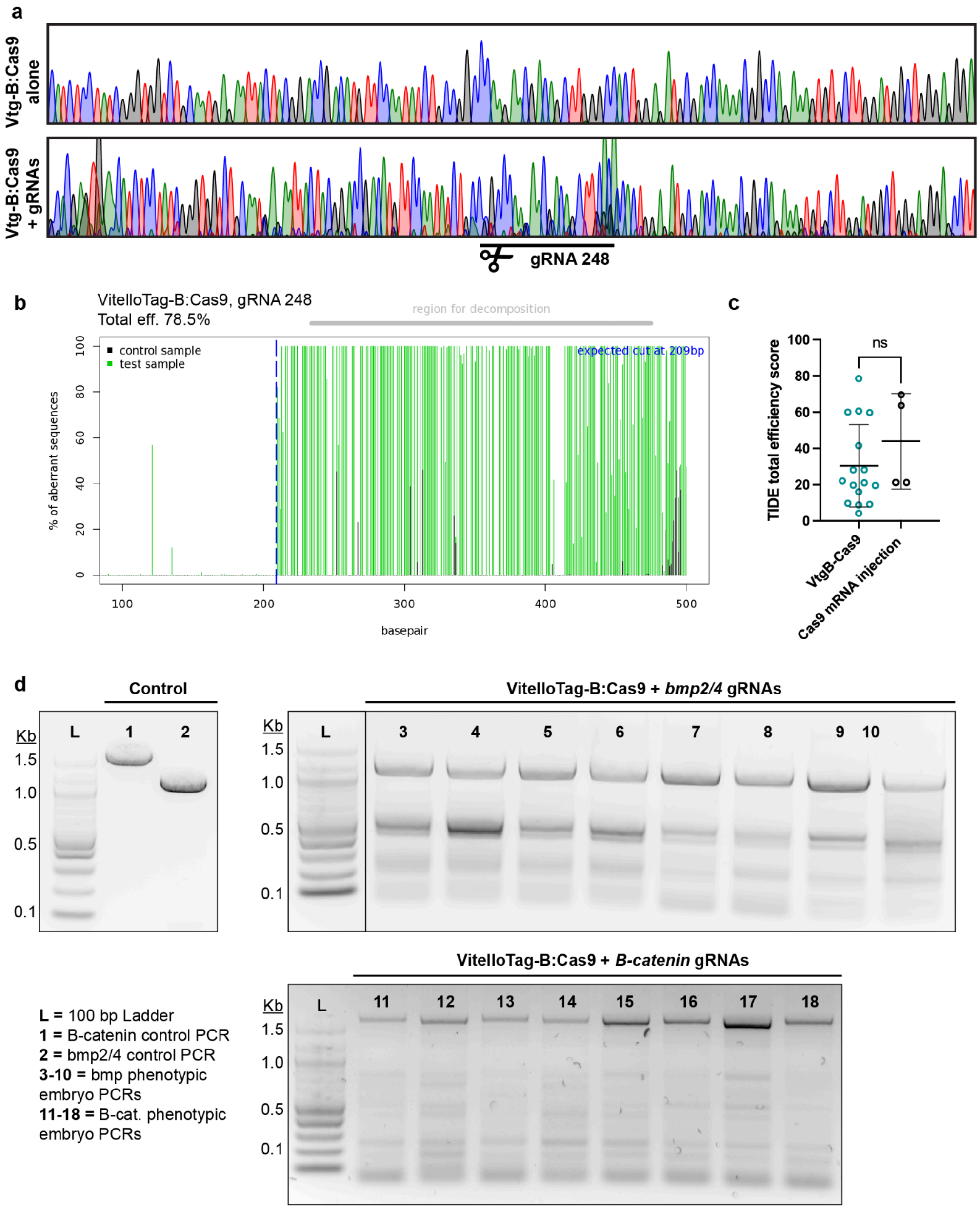

Fig. S6. Incubation with VitelloTag:Cas9 generates indel mutations in *P. miniata* and *S. kowalevskii*.

a. Representative chromatogram from single-embryo genomic PCRs of the *P. miniata delta* locus for embryos treated with VitelloTag-B:Cas9 alone (control) or VitelloTagB + gRNAs.
b. Decomposition plot of TIDE analysis for sequenced PCR products for embryo treated with VitelloTag-B:Cas9 + Delta sgRNAs, relative to VitelloTag-B:Cas9 alone, as shown in (a).
c. Quantification of TIDE total efficiency scores across all embryos analyzed, comparing VitelloTagB:Cas9-treated embryos with embryos injected with Cas9 mRNA. Significance determined by T-test.
d. Agarose gels showing genomic PCR products from single *Saccoglossus* embryos. Full-length, unedited controls (left) are shown in comparison to PCRs from phenotypic *bmp2/4* and *β-catenin* mutants, which show mosaic genomic deletions (right, top and bottom).

Development • Supplementary information

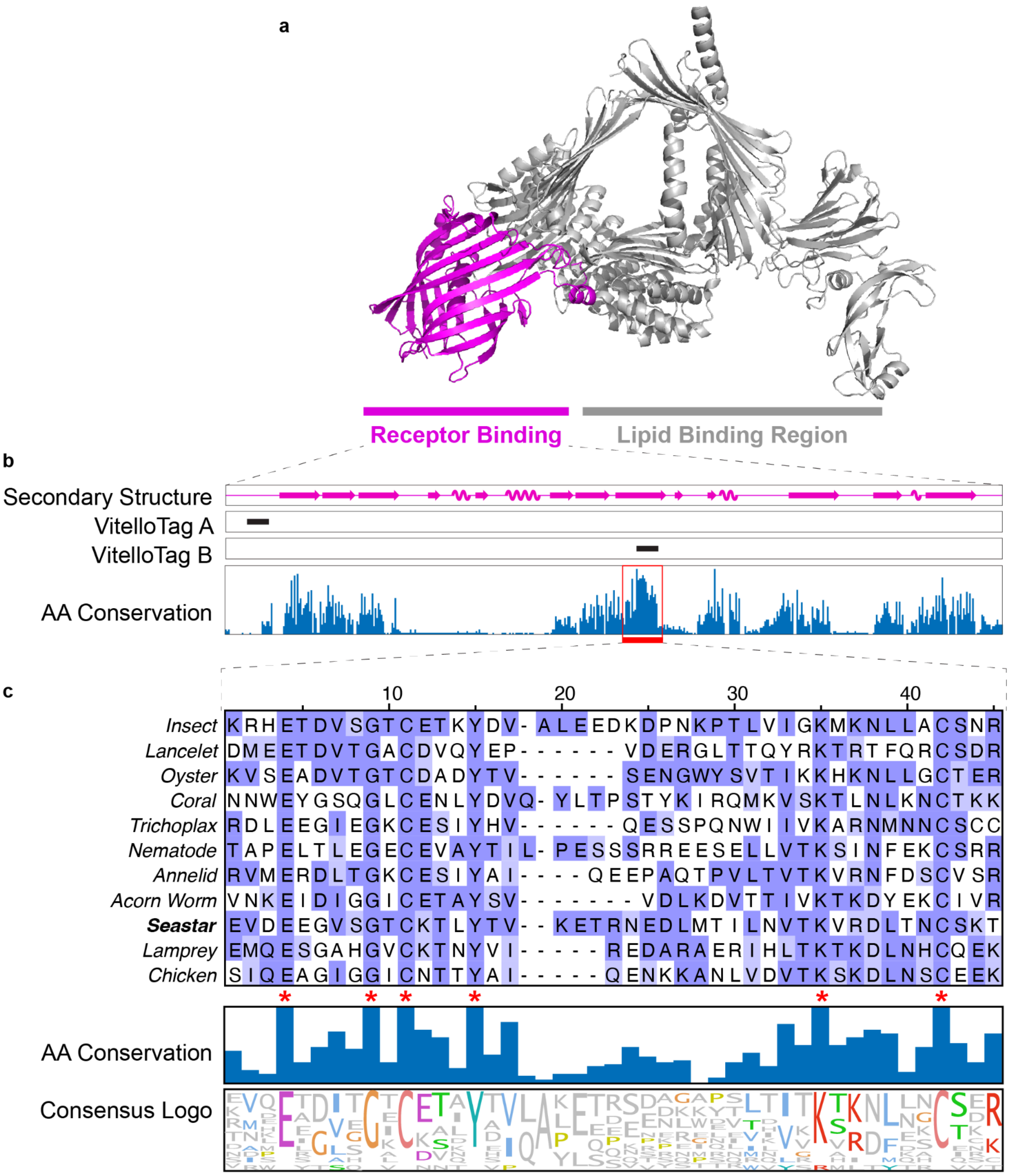

Fig. S7. Sequence conservation analysis of Vitellogenin

a. Crystal structure of Vitellogenin from Lamprey (PDB: 1LSH), showing the receptor binding domain (RBD, magenta).
b. Secondary structure and amino acid conservation of the RBD. Positions of VitelloTag A and B are indicated (black lines). Red box highlights the region shown in (c).
c. Multiple sequence alignment of the VitelloTag-B region across representative species from 9 phyla, including vertebrates. Sequence conservation histogram and consensus logo are shown below. Red asterisks indicate positions with 100% amino acid sequence conservation.

Development • Supplementary information

Table S1. oligonucleotides used in this study

Name	Sequence	Purpose	Comments
PmVtg1_177_F	CCTGTACTTCCAATCCAATgtgtacgaagtggatgaaGAGGGCGTGTCTGGC	cloning	Amplifies Patiria vitellogenin residues 177 to 278
PmVtg1_278_R	ccgttatccacttccaatAGATCCGCCCTTGATGCC	cloning	Amplifies Patiria vitellogenin residues 177 to 278
eSpCas91.1_F	ATGAAAgactataaggaccac	cloning	for amplification of eSpCas91.1
eSpCas91.1_R	ctttttcttttttgcctgg	cloning	for amplification of eSpCas91.1
SkBMP_Ex3_F	CTTGGGAGTCGTTTGATATACGG	genotyping	For genotyping of Saccoglossus Bmp2/4 locus
SkBMP_Ex3_R	GAGCCACTATCCAGTCATTCC	genotyping	For genotyping of Saccoglossus Bmp2/4 locus
SkBcat_Ex3_F	ATTGACCCTCAAGACCTGCC	genotyping	For genotyping of Saccoglossus B-cat. locus
SkBcat_Ex3_R	CAGAAAGCCATTGAACAAGAACC	genotyping	For genotyping of Saccoglossus B-cat. locus
oAK001	ccaaagaagaagcggaaggtcatggataagaaatactcaatag	cloning	Cas9 Forward Primer + SV40 NLS tail
oAK002	tttctttttcttagcttgaccagctttcttagtagcagcaggacgcttgtcacctcctagctgac	cloning	Cas9 Reverse Primer + Nucleoplasmin NLS Tail
oAK003	gaccttccgcttcttctttggAGCCATAGAAGAACCATGG	cloning	SIT Plasmid Reverse Primer + SV40 NLS Overhang
oAK004	agctggtcaagctaagaaaaagaaaATCGAGGAAAACCTGTAC	cloning	SIT Plasmid Forward Primer + Nucleoplasmin Overhang
oAK005	agctggtcaagctaagaaaaagaaaATCGAGGAAAACCTGTAC	cloning	PmVtg196-211 Forward Primer + Nucleoplasmin NLS Overhang
oAK006	gaccttccgcttcttctttggAGCCATAGAAGAACCATGG	cloning	PmVtg196-211 Reverse Primer + SV40 NLS Overhang
oAK007	AGCCATAGAAGAACCATGGTGATGGTGATG	cloning	Reverse Primer to open vector for insertion (SIT Plasmid OR PmVtg196-211 Plasmid)
oAK008	ATCGAGGAAAACCTGTACTTCCAATCCAAT	cloning	Forward Primer to open vector for insertion (SIT Plasmid OR PmVtg196-211 Plasmid)
oZS1032	attgcacACAGGTAGCGAAC	genotyping	For genotyping of Patiria Delta locus; also used for Sanger sequencing
oZS1033	TAAAATGGCTTCCCCTTGCG	genotyping	For genotyping of Patiria Delta locus

Development • Supplementary information

Table S2. Target sequences for gRNAs used in this study

Name	Species	Gene	Target sequence
PmDelta519	*P. miniata*	*delta*	AACGGAGGCACCTGCGAGAA
PmDelta198	*P. miniata*	*delta*	CGGCCCAAGAACGACAGCTT
PmDelta248	*P. miniata*	*delta*	GAAAGTGTGCTTGGATGGCT
SkBmp24-1	*S. kowalevskii*	*bmp2/4*	GAAGAGGCAATCTGATAAAC
SkBmp24-2	*S. kowalevskii*	*bmp2/4*	CTTATAGGACTAAGATCCGT
SkBcat-1	*S. kowalevskii*	*beta-catenin*	CCAAGGAGGATATTATACCC
SkBcat-2	*S. kowalevskii*	*beta-catenin*	AACCTTCTACTTCACCAAGA

Supplementary methods:

VitelloTag:Cas9-gRNA complexing and incubation with oocytes

Margherita Perillo, Akshay Kane, July 2024

1. Collect 100-200 oocytes into 500 μL filtered seawater (FSW) in a 12 well plate.

2. Add the following reagents into a microcentrifuge tube:

Component	Quantity
VitelloTag:Cas9 (without glycerol)	2μL
gRNAs*	0.8 μL each
NEB r3.1 buffer	2 μL
Nuclease free water	make up to 20μL

3. Incubate this mixture for 20 mins at 25°C, followed by 5 mins at 37°C to form Cas9:gRNA ribonucleoprotein (RNP) complexes
4. Add 20μL RNP mixture to well containing oocytes.
5. Add chloroquine (final concentration 20μM) to the well.
6. Incubate at 15°C overnight.
7. Next morning, mature and fertilize oocytes.
8. After fertilization envelope is seen, wash the oocytes after 30 mins with antibiotic-free FSW.

*Note: In case of Cas9 negative control, add nuclease-free water in place of guides.

Development • Supplementary information

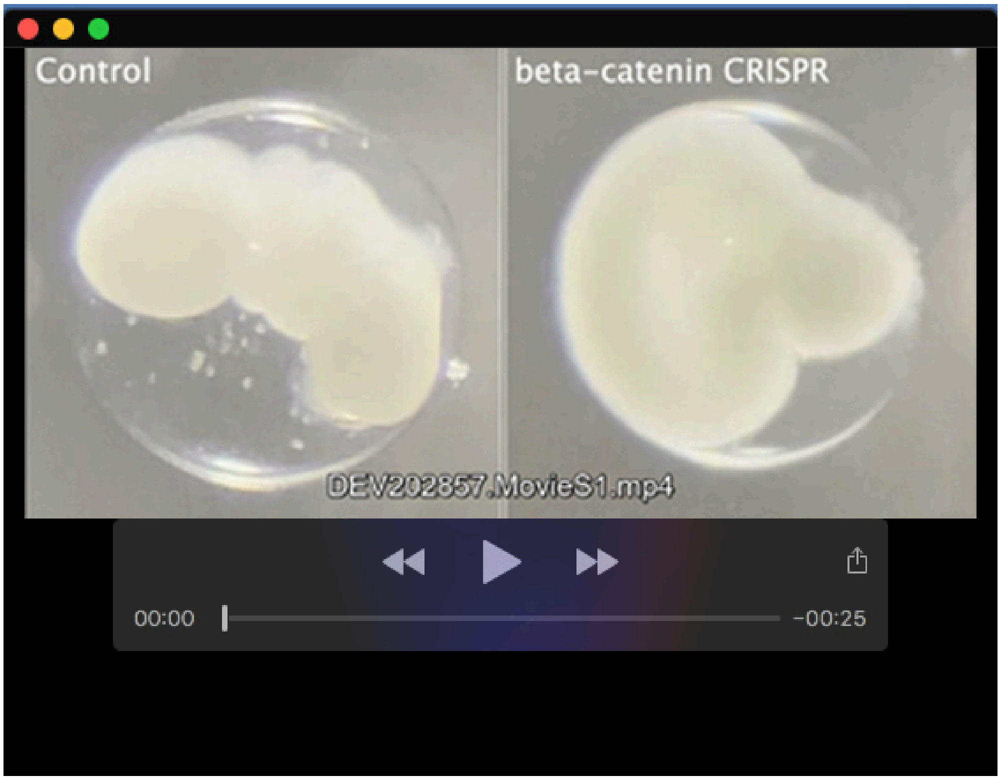

Movie 1. Incubation with VitelloTag:Cas9 RNPs produce zygotic loss-of-function phenotypes in *S. kowalevskii.* In comparison to control oocytes treated with VitelloTag:Cas9 alone (left), oocytes treated with VitelloTag:Cas9 and gRNAs against *beta-catenin* (right) have an anteriorized phenotype, but gastrulate successfully and have a small posterior domain (evident by the small ciliary band), which is consistent with zygotic loss of function. For reference, embryos injected with siRNAs against *beta-catenin* to deplete maternal transcripts (Darras et al., 2011) are fully animalized and do not gastrulate or produce endomesoderm or posterior ectoderm. For both embryos shown, anterior is at left, and dorsal is at top.
