# Supplementary material for: VitelloTag: a tool for high-throughput cargo delivery into oocytes
Source: Development. 2024 Sep 14;151(20):dev202857. doi: 10.1242/dev.202857 (PMC11423919; doi:10.1242/dev.202857)
Supplement: Supplementary file 1 [file develop-151-202857_review_history.pdf]

Development

VitelloTag: a tool for high-throughput cargo delivery into oocytes

D. Nathaniel Clarke, Akshay Kane, Margherita Perillo, Christopher Lowe and S. Zachary Swartz
DOI: 10.1242/dev.202857

Editor: Swathi Arur

Review timeline
Original submission: 5 March 2024
Editorial decision: 8 May 2024
First revision received: 12 August 2024
Accepted: 15 August 2024

Original submission

First decision letter

MS ID#: DEVELOP/2024/202857

MS TITLE: VitelloTag: a tool for high-throughput cargo delivery into oocytes

AUTHORS: Donald Nathaniel Clarke, Akshay Kane, Margherita Perillo, Christopher Lowe, and S. Zachary Swartz

I have now received all the referees reports on the above manuscript, and have reached a decision. The referees' comments are appended below, or you can access them online: please go to BenchPress and click on the 'Manuscripts with Decisions' queue in the Author Area.

The overall evaluation is positive and we would like to publish a revised manuscript in Development. However, each reviewer makes recommendations that can improve the rigor of the analysis. I do not expect to send the paper back to the reviewers, however, I will look over the revision very carefully. Thus, please attend to all of the reviewers' comments in your revised manuscript and detail them in your point-by-point response. If you do not agree with any of their criticisms or suggestions explain clearly why this is so.

Reviewer 1

Advance summary and potential significance to field

The important advance in this study is that it has the potential to be a robust and convenient method widely applicable for efficient gene manipulations and functional studies in important but challenging non-canonical model organisms.
They have demonstrated its ability for generating a high level of gene deletions in a sea star and hemichordate. This hemichordate (Saccoglossus k.) occupies an important phylogenetic position in chordate evolution but functional studies have been challenging. This method is going to facilitate high throughput functional studies that can have a significant impact on our understanding of the evolution developmental and regulatory networks leading to the emergence of vertebrates. This by itself represents an important enough advance to justify publication as a methods paper for studying development and evolution. If the VitelloTag:Cas9 they have generated can be used by an even wider variety of organisms with the only variant being gRNAs for desired targets it could make a big impact in a number of fields.

© 2024. Published by The Company of Biologists under the terms of the Creative Commons Attribution License (https://creativecommons.org/licenses/by/4.0/).

The data seem clear and convincing. The only concerns are whether the vitellogenin Tags they identified will be recognized by receptors in a wide range of species. Its also unknown whether the chloroquine treatments will have adverse effects on development in other species. They show it did not adversely effect processes in the sea star and Saccoglossus but I do not know if that will be the case in other species.

Comments for the author

The paper is clear, well written and the methods sufficiently detailed to enable others to test the approach. If the VitelloTag:GFP were made widely available it would facilitate rapid testing in other species which might help the adoption and validation of this approach in a wider range of species.

One minor correction. line 87 refers to Suppl Fig. 5 but ther is to Suppl Fig. 5.
I think this is a mistake and they mean Suppl Fig.4.

<u>Reviewer 2</u>

Advance summary and potential significance to field

The authors of this manuscript report the development of “VitelloTag” which allows protein cargo to be delivered to oocytes. The tag is a vitellogenin-derived protein, which when fused to a cargo protein, allows the cargo to be taken up into the oocyte via receptor-mediated endocytosis. The authors provide proof-of-principle by introducing GFP into oocytes and then provide a functional example by introducing a Cas9/sgRNA complex into oocytes. Experiments are replicated in two non-chordate deuterostomes, suggesting that this approach may be broadly applied to multiple taxa.

In figure 1, the authors identify functional fragments of vitellogenin that can be used to transport GFP into oocytes. The authors demonstrated that GFP was released from endosomes after chloroquine treatment which is consistent with the expected mode of transit into the oocyte. Subsequently, the authors identified small (10-15 aa) functional vitellogenin fragments that could be used as tags.

In figure 2, the authors demonstrate that Cas9 gene editing reagents can be transported into oocytes using the vitellotag and that these reagents are capable of gene editing events which produce phenotypes consistent for the genes targeted. Gene editing was apparent in both sea stars and hemichordates.

This is a nice technique/reagent development paper which promises to have broad applications if vitellotag can be utilized outside the two animal groups tested.

Comments for the author

I don't have significant suggestions for edits or changes.

Primer sequences and the final peptide sequences of the tags were not included in the review packet so it was not possible to determine if this could apply to other species. Did the authors perform an informatics examination to look for conserved regions in diverse organisms? If so, it might be nice to make some predictions about how broadly this is likely to be functional.

<u>Reviewer 3</u>

Advance summary and potential significance to field

Clarke et al. present a very exciting new tool with enormous potential to revolutionize genetic perturbations across multiple species.

© 2024. Published by The Company of Biologists under the terms of the Creative Commons Attribution License (https://creativecommons.org/licenses/by/4.0/).

Comments for the author

The additional experiments I have requested are crucial in further validating the effectiveness of the protocol and addressing some concerns with the quantification of the results.

The main evidence that the system is working is shown in Figure 2 where the authors show the phenotype of the increased number of mesenchyme cells, which is a feature of delta phenotype in sea stars. However, this number can be very variable normally and is a frequent phenotype shown by unhealthy embryos. It's not a sufficient assay to demonstrate Delta KO. It's also not a good assay of penetrance and mosaicism. There are many great markers of Delta phenotype - including overexpression of gcm in the ectoderm, expression of ets1 in mesenchyme and loss of pax6 expression in the coelomic epithelium. Doing insitu hybridizations in these embryos is very straightforward and would much more convincingly show that they have a delta phenotype. This will also show whether they are getting a full embryo phenotype or getting clonal CAS9 editing as particularly the effect of gcm overexpression would be seen throughout the embryo or only in clones.

The current quantification performed in Fig 2 is not sufficient and not clearly explained. For example,- what does percent phenotypic mean, and how many batches and numbers of embryos were scored? Line 85 - what does strong phenotype mean?

Also, for lines 95 - 99, how was penetrance scored? These experiments should be done with a clear description of replication- e.g., three biological controls of 10 to 20 embryos or however, the authors need to demonstrate the effect. The degree of phenotype (strong etc) should be explained, and representative images should be shown. The authors also need to provide a better description of the TIDE analyses - how many biological replicates and how many embryos were assayed?
Also, please provide some extra text to explain what the Total eff scores mean?
How does this relate to the number of embryos with a KO and the homo-vs heterozygosity of the effect?

These above experiments are critical for demonstrating that they have achieved the cas9 KOs, the degree of the KO, and hence that the system is working.

Figure 1. Is there a better image of GFP localization or can the distribution throughout the cytoplasm be quantified. GFP looks localized to the edge of cell membrane. Are there Z slices that show that GFP is localized throughout and not just near the membrane?

In Suppl Fig 1 C - GFP looks excluded from the nucleus, which is centered within the germinal vesicle. Can you please clarify how the nuclear localization was measured as I do not see this in the images shown, The GFP experiment is very nice and an important first step but it would be very nice also to show localization of CAS9. There should be good working antibodies and the authors, instead of showing the cross sections localization of GFP as I requested above, could instead show the localization of cas9 protein. This would be a much more convincing assay.

The authors should provide an explanation of how the guide RNAs are being uptaken.

Do they require VitelloTag, and/or are they binding to CAS9? Was the protocol designed to bind the guide RNAs and CAS9 invitro prior to uptake? I'm surprised that the guide RNA has also been uptaken, so some additional explanation of how this occurs is needed.

First revision

Author response to reviewers' comments

MS ID#: DEVELOP/2024/202857

Response to reviewers

© 2024. Published by The Company of Biologists under the terms of the Creative Commons Attribution License (https://creativecommons.org/licenses/by/4.0/).

The authors appreciate the constructive comments from the reviewers and the editor. Comments from the reviewers are listed below, each with a specific response that is also reflected in the main text (indicated by line # of the revised text).

General response
We thank all Reviewers for their careful reading of the manuscript, and their thorough and helpful comments and suggestions. The resulting product is strengthened, and we hope that the revisions will be satisfactory for publication.

Point-by-point response to reviewer comments (*reviewers' comments are italicized,* our responses are in blue text)**:**

Reviewer 1 Advance Summary and Potential Significance to Field:
The important advance in this study is that it has the potential to be a robust and convenient method widely applicable for efficient gene manipulations and functional studies in important but challenging non-canonical model organisms. They have demonstrated its ability for generating a high level of gene deletions in a sea star and hemichordate. This hemichordate (Saccoglossus k.) occupies an important phylogenetic position in chordate evolution but functional studies have been challenging. This method is going to facilitate high throughput functional studies that can have a significant impact on our understanding of the evolution developmental and regulatory networks leading to the emergence of vertebrates. This by itself represents an important enough advance to justify publication as a methods paper for studying development and evolution. If the VitelloTag:Cas9 they have generated can be used by an even wider variety of organisms with the only variant being gRNAs for desired targets it could make a big impact in a number of fields.

The data seem clear and convincing. The only concerns are whether the vitellogenin Tags they identified will be recognized by receptors in a wide range of species. Its also unknown whether the chloroquine treatments will have adverse effects on development in other species. They show it did not adversely effect processes in the sea star and Saccoglossus but I do not know if that will be the case in other species.

Reviewer 1 Comments for the Author:
The paper is clear, well written and the methods sufficiently detailed to enable others to test the approach. If the VitelloTag:GFP were made widely available it would facilitate rapid testing in other species which might help the adoption and validation of this approach in a wider range of species.

- Plasmids for VitelloTag:GFP and VitelloTag:Cas9 will be made available on AddGene.

One minor correction. line 87 refers to Suppl Fig. 5 but there is no Suppl Fig. 5. I think this is a mistake and they mean Suppl Fig.4.

- Thank you for catching this. Yes, this is a typo. We have corrected the callout to now refer to Supp. Fig. 6, as we have added additional supplementary figures.

****** Reviewer 2 Advance Summary and Potential Significance to Field:*
The authors of this manuscript report the development of "VitelloTag" which allows protein cargo to be delivered to oocytes. The tag is a vitellogenin-derived protein, which when fused to a cargo protein, allows the cargo to be taken up into the oocyte via receptor-mediated endocytosis. The authors provide proof-of-principle by introducing GFP into oocytes and then provide a functional

© 2024. Published by The Company of Biologists under the terms of the Creative Commons Attribution License (https://creativecommons.org/licenses/by/4.0/).

example by introducing a Cas9/sgRNA complex into oocytes. Experiments are replicated in two non-chordate deuterostomes, suggesting that this approach may be broadly applied to multiple taxa.

In figure 1, the authors identify functional fragments of vitellogenin that can be used to transport GFP into oocytes. The authors demonstrated that GFP was released from endosomes after chloroquine treatment which is consistent with the expected mode of transit into the oocyte.
Subsequently, the authors identified small (10-15 aa) functional vitellogenin fragments that could be used as tags. In figure 2, the authors demonstrate that Cas9 gene editing reagents can be transported into oocytes using the vitellotag and that these reagents are capable of gene editing events which produce phenotypes consistent for the genes targeted. Gene editing was apparent in both sea stars and hemichordates.

This is a nice technique/reagent development paper which promises to have broad applications if vitellotag can be utilized outside the two animal groups tested.

Reviewer 2 Comments for the Author:
I don't have significant suggestions for edits or changes.

Primer sequences and the final peptide sequences of the tags were not included in the review packet so it was not possible to determine if this could apply to other species. Did the authors perform an informatics examination to look for conserved regions in diverse organisms? If so, it might be nice to make some predictions about how broadly this is likely to be functional.

- The lack of primer and peptide sequences was an oversight. We have corrected this mistake with the addition of a supplementary table containing primer sequences used for cloning (Supp. Table 1), and the inclusion of the VitelloTag peptide sequences at line 84. Regarding bioinformatics, we have performed an analysis on the conservation of the VitelloTag regions of Vitellogenin; we have added this as a new supplementary figure (Supp. Fig. 7), and amended the text at lines 141-143 to describe the results of the sequence conservation analysis.

****** Reviewer 3 Advance Summary and Potential Significance to Field:*
Clarke et al. present a very exciting new tool with enormous potential to revolutionize genetic perturbations across multiple species.

Reviewer 3 Comments for the Author:

The additional experiments I have requested are crucial in further validating the effectiveness of the protocol and addressing some concerns with the quantification of the results.

The main evidence that the system is working is shown in Figure 2 where the authors show the phenotype of the increased number of mesenchyme cells, which is a feature of delta phenotype in sea stars. However, this number can be very variable normally and is a frequent phenotype shown by unhealthy embryos. It's not a sufficient assay to demonstrate Delta KO. It's also not a good assay of penetrance and mosaicism. There are many great markers of Delta phenotype - including overexpression of gcm in the ectoderm, expression of ets1 in mesenchyme and loss of pax6 expression in the coelomic epithelium. Doing in situ hybridizations in these embryos is very straightforward and would much more convincingly show that they have a delta phenotype. This will also show whether they are getting a full embryo phenotype or getting clonal CAS9 editing as particularly the effect of gcm overexpression would be seen throughout the embryo or only in

© 2024. Published by The Company of Biologists under the terms of the Creative Commons Attribution License (https://creativecommons.org/licenses/by/4.0/).

clones.

- We appreciate this constructive feedback. We have performed HCR *in situ* hybridizations against *gcm*, as recommended, and added this data to the manuscript in a new supplementary figure (Supp. Fig. 5). We have changed the text at lines 102 -105 to describe the results of these experiments, and the mosaicism based on this experiment as well as TIDE genotyping analysis.

The current quantification performed in Fig 2 is not sufficient and not clearly explained. For example,- what does percent phenotypic mean, and how many batches and numbers of embryos were scored? Line 85 - what does strong phenotype mean? Also, for lines 95 - 99, how was penetrance scored? These experiments should be done with a clear description of replication- e.g., three biological controls of 10 to 20 embryos or however, the authors need to demonstrate the effect.

- We have added a description of how phenotypic embryos were quantified to the Methods section at lines 262-264. Regarding 'strong phenotype', please see our response to the following comment. We have added a description of how penetrance was scored to the Methods section at line 280.

The degree of phenotype (strong etc) should be explained, and representative images should be shown.

- To avoid confusion, we have removed the qualifying word 'strong' as a descriptor from this section of the text. In our quantification, we only characterize embryos as either 'phenotypic' or wildtype, so saying 'strongly phenotypic' incorrectly implies that we scored embryos into multiple gradations of phenotypes (e.g., strong vs. weak). Regarding quantification of phenotypes, please see our response to the previous comment.

The authors also need to provide a better description of the TIDE analyses - how many biological replicates and how many embryos were assayed?

- We have added a brief description of the TIDE analysis to the text at line 105-108, and also added an additional plot to Supp. Fig. 6 that summarizes the entirety of the TIDE analyses we performed. In total, the current analysis includes 16 Vtg-Cas9 embryos across 3 replicates with oocytes from 3 different females, and 1 replicate of 4 Cas9 mRNA injected embryos (this is also summarized in the figure legend).

Also, please provide some extra text to explain what the Total eff scores mean? How does this relate to the number of embryos with a KO and the homo-vs heterozygosity of the effect?

- We have added a definition of Total Efficiency Score to the main text at line 107.

These above experiments are critical for demonstrating that they have achieved the cas9 KOs, the degree of the KO, and hence that the system is working.

Figure 1. Is there a better image of GFP localization or can the distribution throughout the cytoplasm be quantified. GFP looks localized to the edge of cell membrane. Are there Z slices that show that GFP is localized throughout and not just near the membrane?

- As noted by the reviewer, endocytosed vesicles are predominantly localized to the cell periphery. We have amended the text to better describe this localization (line #58). It

© 2024. Published by The Company of Biologists under the terms of the Creative Commons Attribution License (https://creativecommons.org/licenses/by/4.0/).

is important to note that for the data in Fig. 1, the oocytes are not treated with endosome escape reagents, and therefore the GFP signal should be restricted to endosomes and not ubiquitous throughout the cell.

In Suppl Fig 1 C - GFP looks excluded from the nucleus, which is centered within the germinal vesicle. Can you please clarify how the nuclear localization was measured as I do not see this in the images shown,

- In this analysis, the signal being measured is the diffuse signal, not the puncta. The GFP intensity was specifically measured within an ROI in the nucleus, which we have described in the methods section. We have also added text to the figure legend for this panel to clarify this.

The GFP experiment is very nice and an important first step but it would be very nice also to show localization of CAS9. There should be good working antibodies, and the authors, instead of showing the cross sections localization of GFP as I requested above, could instead show the localization of cas9 protein. This would be a much more convincing assay.

- As requested by the reviewer, we performed an immunostaining to show the localisation of Cas9 in the cytoplasm and nucleus of oocytes incubated with VitelloTagB:Cas9. We have compared these oocytes to ones injected with Cas9 mRNA, as well as a control group treated with neither. We have added a description of the results of these experiments to the text at lines 89-93, and include this data in a new supplementary figure (Supp. Fig. 4).

The authors should provide an explanation of how the guide RNAs are being uptaken. Do they require VitelloTag, and/or are they binding to CAS9? Was the protocol designed to bind the guide RNAs and CAS9 in vitro prior to uptake? I'm surprised that the guide RNA has also been uptaken, so some additional explanation of how this occurs is needed.

- Vtg-Cas9:gRNA ribonucleoprotein complexes were formed prior to addition to oocytes. This methodology is well established, and has been shown to be sufficient to enable gRNA co-transport in other transfection-based CRISPR/Cas9 genome editing approaches (e.g., DeWitt et al., 2017, PMID: 28410976). We have added additional language to the Methods section at line 256 to clarify that we are pre-forming RNP complexes.

<u>Second decision letter</u>

MS ID#: DEVELOP/2024/202857

MS TITLE: VitelloTag: a tool for high-throughput cargo delivery into oocytes

AUTHORS: Donald Nathaniel Clarke, Akshay Kane, Margherita Perillo, Christopher Lowe, and S. Zachary Swartz

I am happy to tell you that your manuscript has been accepted for publication in Development, pending our standard publication integrity checks.

© 2024. Published by The Company of Biologists under the terms of the Creative Commons Attribution License (https://creativecommons.org/licenses/by/4.0/).
